# The Use of Natural Language Processing Methods in Reddit to Investigate Opioid Use: Scoping Review

**DOI:** 10.2196/51156

**Published:** 2024-09-13

**Authors:** Alexandra Almeida, Thomas Patton, Mike Conway, Amarnath Gupta, Steffanie A Strathdee, Annick Bórquez

**Affiliations:** 1 Scientific Computing Program Oswaldo Cruz Foundation Rio de Janeiro Brazil; 2 San Diego State University School of Social Work San Diego, CA United States; 3 Department of Medicine University of California San Diego San Diego, CA United States; 4 School of Computing and Information Systems The University of Melbourne Melbourne Australia; 5 San Diego Supercomputer Center University of California San Diego San Diego, CA United States

**Keywords:** opioid, Reddit, natural language processing, NLP, machine learning

## Abstract

**Background:**

The growing availability of big data spontaneously generated by social media platforms allows us to leverage natural language processing (NLP) methods as valuable tools to understand the opioid crisis.

**Objective:**

We aimed to understand how NLP has been applied to Reddit (Reddit Inc) data to study opioid use.

**Methods:**

We systematically searched for peer-reviewed studies and conference abstracts in PubMed, Scopus, PsycINFO, ACL Anthology, IEEE Xplore, and Association for Computing Machinery data repositories up to July 19, 2022. Inclusion criteria were studies investigating opioid use, using NLP techniques to analyze the textual corpora, and using Reddit as the social media data source. We were specifically interested in mapping studies’ overarching goals and findings, methodologies and software used, and main limitations.

**Results:**

In total, 30 studies were included, which were classified into 4 nonmutually exclusive *overarching goal* categories: methodological (n=6, 20% studies), infodemiology (n=22, 73% studies), infoveillance (n=7, 23% studies), and pharmacovigilance (n=3, 10% studies). NLP methods were used to identify content relevant to opioid use among vast quantities of textual data, to establish potential relationships between opioid use patterns or profiles and contextual factors or comorbidities, and to anticipate individuals’ transitions between different opioid-related subreddits, likely revealing progression through opioid use stages. Most studies used an embedding technique (12/30, 40%), prediction or classification approach (12/30, 40%), topic modeling (9/30, 30%), and sentiment analysis (6/30, 20%). The most frequently used programming languages were Python (20/30, 67%) and R (2/30, 7%). Among the studies that reported limitations (20/30, 67%), the most cited was the uncertainty regarding whether redditors participating in these forums were representative of people who use opioids (8/20, 40%). The papers were very recent (28/30, 93%), from 2019 to 2022, with authors from a range of disciplines.

**Conclusions:**

This scoping review identified a wide variety of NLP techniques and applications used to support surveillance and social media interventions addressing the opioid crisis. Despite the clear potential of these methods to enable the identification of opioid-relevant content in Reddit and its analysis, there are limits to the degree of interpretive meaning that they can provide. Moreover, we identified the need for standardized ethical guidelines to govern the use of Reddit data to safeguard the anonymity and privacy of people using these forums.

## Introduction

### Background

Opioid use disorder (OUD) is a chronic condition that affects more than 40 million people worldwide [1]. In 2019, the Global Burden of Disease study estimated that 128,000 deaths were attributed to drug use disorders [2], and the United States accounted for a large proportion of these deaths, with >70,000 deaths directly attributable to overdose [3]. US overdose deaths reached a record high in 2021, with 107,000 deaths, of which 75% were opioid related [4]. The magnitude of these numbers highlights the need for a strong surveillance infrastructure, including systems that support tracking trends in drug use and emerging patterns in the drug supply chain, as well as a good understanding of the experiences of, challenges of, and sources of support for people who use opioids, to inform a timely and effective response to this ongoing epidemic.

Social media platforms represent an important and accessible source of community support for people who use opioids, given they facilitate getting technical know-how and peer support. Nonetheless, analyzing such large textual data sets is challenging. Beyond the traditional qualitative approach to analyzing textual data, natural language processing (NLP) allows the analysis of textual data using computational methods and artificial intelligence. NLP lexicon-based methods and both supervised and unsupervised machine learning tools have been used to explore substance-related research questions on platforms such as Twitter (subsequently rebranded X; X Corp) [5-10], Reddit (Reddit Inc) [11-13], and web-based health communities [11,13].

In contrast with Reddit, which allows for long-form narratives, Twitter provides very condensed information for each data point since, historically, posts have been limited to 280 characters. An additional characteristic of tweets as a data type is that (at least in some cases) tweets contain metadata geolocational information, allowing for the ecological study of associations between the volume of mentions of a particular topic and a health outcome of interest in a given setting [14,15]. Facebook (Meta Platforms Inc) offers the advantage of providing information on social networks and the dissemination of information within these networks [16]. However, Facebook data are not necessarily public in the same way that Twitter and Reddit data are public (ie, on the open web). Facebook users typically form closed and semiprivate communities in which there is an expectation of data privacy, rendering the data unsuitable (both practically and ethically) for research purposes, as a specific consent model and process is absent. Reddit is a publicly accessible social media platform, with forums created and moderated to discuss specific themes. Participants (ie, redditors) use the platform anonymity and “throwaway” user accounts to share news, content, and thoughts through posting. Redditors’ anonymity is key to authentic accounts of both positive and negative experiences with drugs (including in the context of treatment) and daily life situations that may impact the physical and mental health of people who use opioids, without fearing stigmatization. This platform is one of the most popular web-based social media platforms and has provided a space for exchange and discussion since 2005. It is mainly used by English speakers [17].

Despite Twitter being the platform of choice for most studies [18], Reddit thematic forums have shown to be an advantage for research on substance use, skipping the filtering stage of posts related to the research topic. NLP techniques enable the use of massive text corpora and the exploration of a multitude of research questions related to opioid use based on the perspectives of people sharing their experiences on social media platforms, such as Reddit.

Reddit ranked as the third most visited website in the United States [19], and in October 2020, it achieved >52 million daily active users [20]. Reddit has been used as a source of information to understand drug-related epidemics mainly due to the lower risk of social desirability bias, the real-time aspect of the data, and lower noise due to its thematic forums structure [21-23]. Given that the majority of its users are from the United States (47.82%), United Kingdom (7.6%), Canada (7.45%), and Australia (3.89%) and that these countries are all undergoing severe opioid-related epidemics, Reddit represents a valuable source of data to better understand the daily experiences of people who use opioids in these settings [24].

### Objective

This scoping review aimed to understand how Reddit data have been leveraged to study the opioid epidemic using NLP to provide an overview of how these novel data sources and tools can serve the field of substance use research. More specifically, we were interested in mapping the studies’ (1) overarching goals and findings, (2) methodologies and software used, and (3) main limitations. In addition, to help future research in this area to deal with abbreviations, slang, and common misspellings, we systematically collected the papers’ list of synonyms and built a comprehensive synset with semantically equivalent terms within the opioid use field. This list could help the broader research community to rigorously investigate opioid use in Reddit using text processing algorithms.

## Methods

### Overview

This scoping review followed the Joanna Briggs Institute (JBI) methodology for scoping reviews based on the studies by Arksey and O’Malley [25] and Levac et al [26]. We used the JBI population (or participants)/concept/context framework to guide our research questions and ultimately inform our search strategy to understand how NLP methods have been applied to Reddit data to inform research on opioid use. Our study protocol was registered on the Open Science Framework [27] on June 29, 2022. Multimedia Appendix 1 details the JBI population (or participants)/concept/context framework rationale behind each research question of this scoping review.

### Search Strategy

As the research topic encompasses studies targeting opioid use, we selected the following search engines: (1) PubMed to cover the biomedical literature; (2) Scopus to cover life, social, physical, and health sciences; (3) PsycINFO to cover the psychology field; and (4) ACL Anthology to cover NLP and computational linguistics. As the research team was aware of recent studies using NLP in the substance use field led mainly by authors from the engineering field, we also included IEEE Xplore and Association for Computing Machinery as data repositories for conference abstracts.

Our search was built based on team members’ expertise in the topic and aimed to identify studies jointly covering three essential aspects: (1) opioid use, as the subject under analysis; (2) NLP techniques, as the methods used to analyze the textual corpora; and (3) Reddit, as the data source used in the study’s analyses. The terms used to capture opioid-related studies were based on standard terms describing opioids, including “heroin” and “fentanyl” as well as the National Institute on Drug Abuse’s list of most commonly prescribed opioids [28] and medications for OUD (MOUD) [29]. To ensure our search would only select studies that used a quantitative methodology to analyze the textual data (ie, NLP), we leveraged the “artificial intelligence” methods included in the Medical Subject Headings vocabulary thesaurus [30], which we supplemented with members’ expertise in the topic to broaden our scope. As many essential papers in this field do not state “Reddit” as the social media environment under analysis in the abstract, and many search engines only consider the abstract (and not the full paper), we included the broad term “social media” in the search. Multimedia Appendix 2 details the search strategy, presenting the Medical Subject Headings hierarchy and the final search query used to obtain relevant studies.

We included papers that appeared in the search until July 19, 2022, with no cutoff for earlier studies. We selected original research papers published in English that stated that they collected textual data on opioid use from Reddit forums (even if other social media were also used) and specified they used NLP techniques to analyze these data.

Our focus was on studies that applied quantitative techniques to textual data. NLP can use a variety of different methods, including rule-based methods (eg, linguistic rules, deployment of lexicons, and development of bespoke regular expressions) and machine learning–based methods (eg, supervised classification and sequence labeling–based methods and unsupervised methods). Therefore, studies that used manual annotation were only included if they also applied quantitative methods to analyze the textual data.

Two researchers screened papers considering the title and abstract. If opioid use was not stated but the authors investigated substance use (eg, “...we evaluated attitudes of people who use substances*...*”), we included the papers for the second round of screening. If the methodology indicated any attempt to use NLP in any step of the study or if the authors were not specific about using only qualitative techniques (eg, “...we manually classified a set of posts*...*”), we included the paper. Finally, if the paper did not mention Reddit but was not specific about using another social media platform, we included it (eg, papers saying “...we evaluated Twitter data...” were excluded, whereas papers stating “...we used social media discussions to understand...” were included). The full-text review excluded the following: (1) review papers (to avoid double-counting); (2) papers solely using qualitative techniques or approaching substance use in general without any particular (or stratified) analysis for opioid-related information; and (3) papers not using Reddit as the data source. AA and TP independently screened for the title, abstract, and full paper. In both stages, when AA and TP diverged in their opinions about the papers’ eligibility, AB (a third reviewer blinded to their classification) decided on eligibility as a tiebreaker. Each reviewer was responsible for the data extraction of critical outcomes of interest.

### Data Extraction

We systematically extracted the following information: study’s year of publication; first author’s country of affiliation; authors’ areas of expertise; social media platforms investigated (in addition to Reddit); subreddit investigated; period under analysis; types of posts analyzed; number of posts analyzed; number of Reddit users represented; whether the analysis was limited to opioids and whether it distinguished between different types of opioids to infer the scope of the population investigated; whether the study attempted to have a geographic focus; whether the study described their semantic analysis to identify opioid-related terms and whether they provided a synset; study’s overarching goal, objectives, main findings, limitations, NLP methods and software used; and ethical approval information.

We followed the PRISMA-ScR (Preferred Reporting Items for Systematic Reviews and Meta-Analyses Extension for Scoping Reviews) [31] guidelines to ensure methodological quality and clarity of the findings. The detailed PRISMA-ScR checklist describing the 20 essential and 2 optional reporting items is presented in Multimedia Appendix 3.

### Semantic Analysis and “Opioid Synset” Development

To help further studies more efficiently address opioid-related research questions using social media data, we identified the different strategies used to carry out semantic analyses of opioid-related content, compiled their lists of opioid-related terms (produced either by automatic variant generator tools or by word embedding) or similar words, and curated their content, creating a refined version where the terms are aggregated by abbreviation, misspelling, variants and their misspellings, brand names and their misspellings, slang, analogues, and mixed substances.

## Results

### Selection of Sources of Evidence

**Figure 1:**
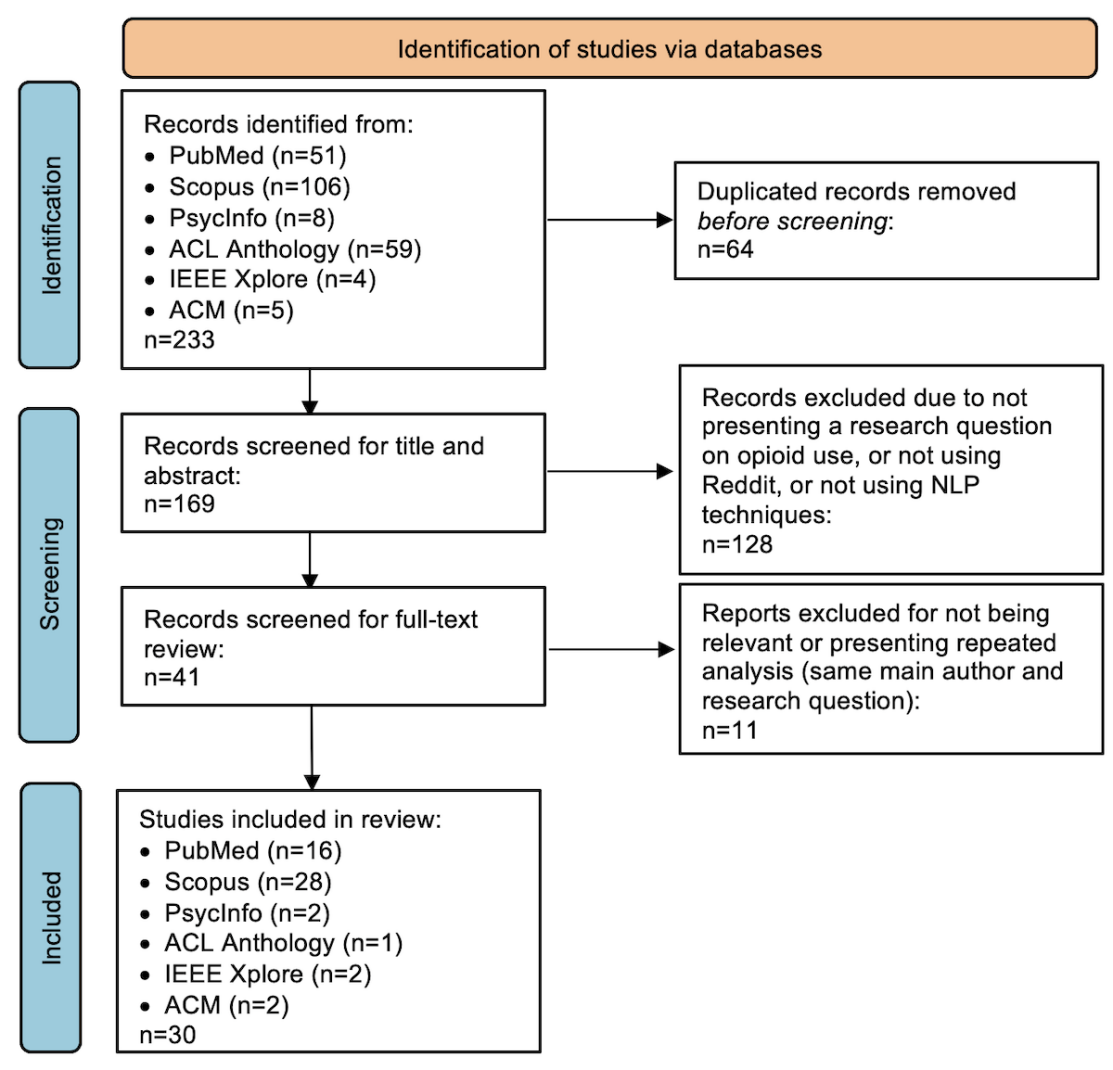
Diagram of the PRISMA (Preferred Reporting Items for Systematic Reviews and Meta-Analyses)–oriented review process. ACM: Association for Computing Machinery; NLP: natural language processing. Note: Under “Studies included in Review,” some of the papers were available on more than one database.

The literature search returned 233 papers, of which 64 (27.4%) were duplicates, 128 (54.9%) were considered ineligible and were thus removed after the title and abstract screening, and 11 (4.7%) were similarly removed after the full-text screening, resulting in a final list of 30 papers. Figure 1 displays the search process, following PRISMA (Preferred Reporting Items for Systematic Reviews and Meta-Analyses) guidelines [32]. A table with a summary of all study findings is provided in Multimedia Appendix 4. Graphs illustrate the main results to give an overview of the literature studied.

### Studies’ Characteristics

As detailed in Table 1, the number of studies applying NLP methods to data from Reddit forums to study opioid use has increased over time. Over half of the studies (19/30, 64%) were published in peer-reviewed journals, over a third (10/30, 33%) were published in conference proceedings, and 1 (3%) was published as a chapter in a book. Most studies (28/30, 93%) were disseminated in platforms with subject areas in computer science and health. Half of the studies (16/30, 53%) were exclusively concerned with opioids and OUD, while the remaining studies (14/30, 47%) were broader in scope (eg, substance use). However, content relating to other substances was sometimes referenced in the studies focused on opioids. For instance, the study by Preiss et al [33] identified a broader range of substances used for self-medication among people experiencing opioid withdrawal symptoms.

**Table 1:** Summary of characteristics of studies using natural language processing methods to investigate opioid use (N=30).

Study characteristics				Studies, n (%)
**Year of publication**				
	2017			1 (3)
	2018			1 (3)
	2019			5 (17)
	2020			6 (20)
	2021			5 (17)
	2022			12 (40)
**Dissemination platform**				
	Peer-reviewed journal			19 (64)
	Conference proceedings			10 (33)
	Book chapter			1 (3)
**Subject area of the dissemination platform**				
	Computer science			10 (33)
	Health and medicine			8 (27)
	Both computer science and health and medicine			10 (33)
	Other			2 (7)
**Range of substances considered**				
	Limited to opioids			16 (53)
	Distinction between different opioid types			11 (37)
**Software used for analysis^a^**				
	Not reported			6 (20)
	**Python**			20 (66)
		The Python Reddit API Wrapper	11 (37)	
		Gensim	4 (13)	
		spaCy	3 (10)	
	Linguistic Inquiry and Word Count			2 (7)
	R			2 (7)
	IBM Watson Natural Language Understanding			1 (3)
**Reported sources of research funding^b^**				
	Financial support not reported			8 (27)
	National Science Foundation			7 (23)
	National Institute on Drug Abuse			5 (17)
	Other (eg, institute-specific funding)			4 (13)
	Other institutes within the National Institutes of Health			4 (13)
	Centers for Disease Control and Prevention			3 (10)
	No funding associated with the study			2 (7)
	Natural Sciences and Engineering Research Council of Canada Discovery Grant			1 (3)

^a^Some studies used multiple software packages.

^b^Some studies had multiple research grants.

Over a third (11/30, 37%) of the reviewed studies explicitly referred to specific opioid types in the research methodology. The content under analysis varied as some studies focused on specific elements of posts, such as the title, the first submission, or the comments, with most of the papers that explained the unit of analysis using a combination of the first submission and comments (Multimedia Appendix 4). Python was the most common programming language used to analyze posting content, although alternative analysis methods and languages were also reported, including R, IBM Watson’s Natural Language Understanding application, and Linguistic Inquiry and Word Count software.

In total, 8 (N=30, 27%) studies did not report any sources of funding and another 2 (7%) studies reported that there was no funding associated with the research. Among the remaining studies, the most common source of funding was the National Science Foundation (7/30, 23% of studies) followed by the National Institute on Drug Abuse (5/30, 17% of studies). Furthermore, 5 sponsored grants were found to have supported multiple studies in the review. Table 2 describes the authors from studies identified in the review, almost all (99/104, 95%) of whom were based in North America. The most common field of education among the authors, based on their terminal degree, was computer science (43/104, 41%). The studies analyzed more than 20 subreddits, most of which covered specific drugs and treatments, such as r/methadone and r/suboxone. However, the rooms with a broader scope and higher number of redditors, such as r/opiates and r/opiatesrecovery, were more frequently used as data sources, in 19 (63%) and 18 (60%) studies, respectively (Multimedia Appendix 4).

**Table 2:** Summary of researcher characteristics authoring studies using natural language processing methods to investigate opioid use (N=104).

Researcher characteristics			Researchers, n (%)
**Number of studies per researcher**			
	1	90 (86.5)	
	2	9 (8.7)	
	4	5 (4.8)	
**Location**			
	United States	92 (88.5)	
	Canada	7 (6.7)	
	Italy	4 (3.8)	
	Multiple locations (Australia and the United States)	1 (0.9)	
**Area of expertise (based on terminal degree)**			
	Computer science	43 (41.3)	
	Medicine	12 (11.5)	
	Health-related (eg, public health)	11 (10.6)	
	Unclear	8 (7.7)	
	Data science or analytics or machine learning	5 (4.8)	
	Multidisciplinary	5 (4.8)	
	Psychology	5 (4.8)	
	Engineering	5 (4.8)	
	Mathematics and statistics	3 (2.9)	
	Physics	2 (1.9)	
	Biology	1 (0.9)	
	Chemistry	1 (0.9)	
	Linguistics	1 (0.9)	
	Sociology	1 (0.9)	
	Social work	1 (0.9)	

### Overarching Goal, Methods Used, and Main Findings

To broadly characterize opioid-related research using Reddit data, we classified the studies according to their overarching goal. We divided them into four groups based on their contribution to the field: (1) methodological, (2) infodemiology, (3) infoveillance, and (4) pharmacovigilance. Methodological papers proposed novel NLP methods to analyze Reddit data or provide scientific tools. They apply these methods or tools to opioid use as an illustrative example but are not focused on this public health problem. Infodemiology, as defined by Eysenbach [34], is a science analogous to traditional epidemiology but with information coming from electronic media and with the ultimate goal of informing public health actions. Infoveillance papers contemplate the “longitudinal tracking of infodemiology metrics for surveillance and trend analysis” [35]. Pharmacovigilance covers the detection of new drugs and the surveillance of drugs and their adverse effects [36]. The papers’ classification followed the procedures adopted in the study’s screening process: AA and TP independently classified the papers according to their overarching goal, and any divergence was discussed and resolved by AB as a tiebreaker.

**Figure 2:**
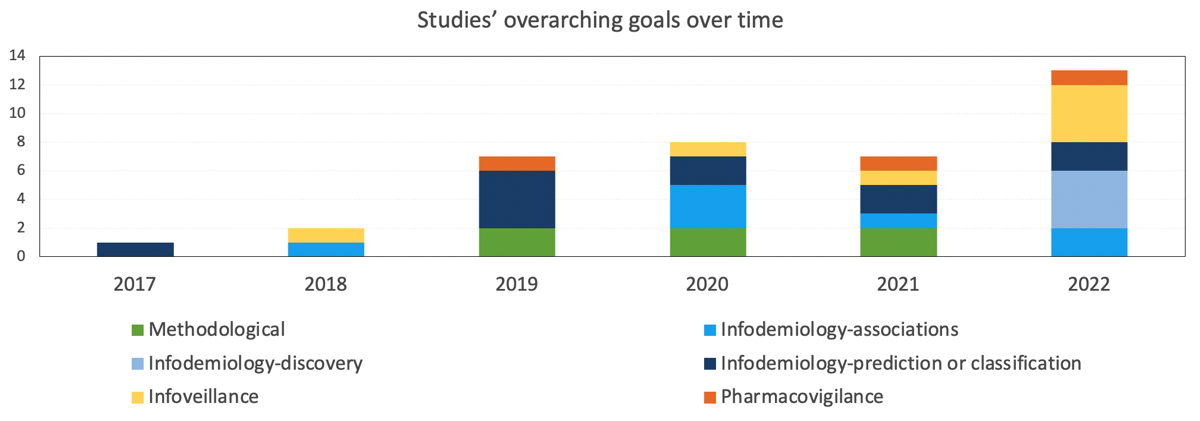
Number of studies investigating opioid use per year using Reddit data and studies’ overarching goals (each study could address more than 1 overarching goal).

The number of studies published each year by overarching goals is presented in Figure 2, where we can see the increased volume and diversity of overarching goals over time. Studies could address more than one overarching goal; therefore, the cumulative number of studies represented is higher than the total number of studies included in the review.

### Methodological Studies

In total, 6 (20%) studies were classified as methodological studies. Adams et al [37] compared Reddit and Twitter as sources of social media information relevant to opioid use and to improve keyword synonym lists for drug-term exploration using embedding techniques. They found that Reddit is better than Twitter as a source for discovering synonyms to be included in a keyword filter list for opioids. Akioyamen et al [38] provided ways for users to navigate Reddit posts to find the content of their interest using topic modeling and showed that text mining could play a fundamental role in supporting the identification of similar documents. Davis et al [39] proposed an archetype-based modeling and search method to identify messages from people who use opioids through learning from their usual vocabulary, which solicits documents from a user and finds similar documents posted by new authors based on the vocabulary. Jha and Singh [40] used a set of NLP techniques, including topic modeling, sentiment analysis, and propensity score matching to extract, treat, and analyze social media textual data on legal and illegal drugs to make it available to the academic community through a web application called SMARTS. Wright et al [19] developed new measures to detect changes in words’ meaning and their association with overdose by applying word embedding and neural network techniques and found that the semantic proximity of fentanyl moved more closely to overdose and overdose-related terms over time. Zhu and Bhat [41] proposed a novel method to identify euphemisms or nicknames for drugs using single and multiwords embedding techniques. The new approach, known as euphemistic phrase detection, was reported to have higher detection accuracies compared to alternative methods.

### Infodemiology Studies

Of the 22 studies classified as infodemiology, 7 (31%) explored associations: Andy [42] investigated correlations between different types of self-disclosures (positive and negative) and social support seeking (emotional and informational). The results showed that correlations between positive self-disclosure and emotional support seeking were moderate to strong, while those between negative self-disclosure and informational support seeking were low to moderate. Balsamo et al [43] estimated the odds ratio between opioid use and (1) routes of administration and (2) drug-tampering methods and routes of administration and drug-tampering procedures. Their findings suggest that substances were consumed in multiple, nonexclusive ways. Jha and Singh [44] explored associations between various latent constructs, addiction recovery, and relapse (defined as posted by users in drug addiction recovery [DAR] forums). Latent constructs capturing emotional distress, physical pain, self-development activities, and social relationships were all significantly associated with addiction recovery (ie, if a user posted in a DAR subreddit). Latent constructs capturing social activities and physical exercise were significantly associated with addiction relapse (ie, if a user posted in a recreational drug use subreddit after posting in a DAR subreddit).

Spadaro et al [45] explored associations between buprenorphine induction, fentanyl use, and precipitated opioid withdrawal. The paper found a relationship between increased mentions of fentanyl and its analogues with (1) increased mentions of precipitated opioid withdrawal and (2) increased mentions of the Bernese method (a microdosing strategy for buprenorphine induction). Pandrekar et al [46] evaluated the association between posts of social support (measured by the number of comments and the difference between upvotes and downvotes received) and the attributes of those posts, including the use of specific terms, the semantic categories observed, and posts’ length. These analyses suggested that posts on personal issues like family, death, and home are positively associated with increased social support. Pandrekar et al [46] also explored the differences between posts from anonymous and nonanonymous users, with the former being more likely to discuss negative emotions like anxiety and sadness and containing words related to health and risk. Andy and Guntuku [47] explored the relationship between social support and the number of comments received using a mix of topic modeling and sentiment and correlation analysis. Their results showed that the average number of comments received was (1) positively associated with emotional support seeking and (2) negatively associated with information support seeking. Finally, Alambo et al [48] explored topical correlations between postings on substance use disorders and COVID-19 between May 2020 and Sep 2020. The results showed that these correlations fluctuated over time.

In total, 4 (18%) studies used discovery techniques to unveil patterns in the data. Ramachandran et al [49] used sentiment analysis and ANOVA to explore the public perceptions of the opioid epidemic. The results showed an overall negative sentiment with slight variation in emotional tones across different subreddits. Gauthier et al [50] tried to understand how Reddit communities support recovery using a topic-guided thematic analysis. The findings showed that community members often raised concerns about sensitive issues, such as withdrawal symptoms, body weight, legal troubles, and personal finances, and they encouraged others to seek help and navigate 12-step programs. Chen et al [51] used topic modeling to explore stigma-related experiences associated with substance use. This study found topics related to negative feelings, the challenges of coping with withdrawal symptoms, dealing with others during the recovery process, and dealing with chronic pain. Graves et al [52] used manual annotation and lexical similarity filter to yield information about firsthand experiences with buprenorphine-naloxone. They showed that the most frequently discussed topics included advice on the use of buprenorphine-naloxone as pharmacological treatment for OUD, information and guidance on dosage, information about tapering of dosage, its side effects and withdrawal information, and specific questions about use.

In total, 11 (50%) infodemiology studies targeted the prediction or classification of people along the OUD continuum. A common theme in these studies was the prediction of redditors’ transitions between drug use, help seeking, and recovery stages. Furthermore, 2 (18%) studies developed models to predict whether redditors would post in a recovery subreddit based on their nonrecovery subreddit activities [53,54]. Another 2 (18%) studies developed models to predict relapse, albeit in different ways. Jha et al [55] predicted the likelihood of changes in the content of users’ posts indicative of relapse. Yang et al [56] predicted substance use relapse in the following week using manually labeled data to identify instances where redditors self-reported experiencing a relapse. Relapse prediction was based on emotions detected in redditors’ previous posting activities.

Among the remaining prediction or classification studies, 4 (36%) assessed the predictive performance of classification models for sorting Reddit content into groups. Davis et al [39] compared various classification models to predict whether redditors’ posts indicated opioid use and found that the linear support vector machine model yielded the best performance. Yao et al [57] developed 2 sets of classifiers aiming to extract suicide risk posts from an opioid use context and extract opioid addiction posts from a suicidal ideation context. The former analysis yielded results showing (1) prediction accuracy and optimal model specification of classifiers varying with the percentage of suicide risk labels and (2) classifiers with better accuracy compared to those extracting opioid addiction posts from a suicidal ideation context. Chancellor et al [58] developed a classification model to determine whether a post was about OUD recovery. Another study [59] presented two classifiers that (1) sorted redditors into OUD and non-OUD groups according to their posting content and (2) determined whether those in the OUD group exhibited evidence of being in a positive recovery process. ElSherief et al [60] developed a classifier to identify posts related to a common myth that using MOUD is simply replacing one drug with another, which may discourage people from seeking this evidence-based treatment.

A study by Andy [42] assessed the predictive performance of models developed to measure the degree of positive and negative self-disclosure of post titles. The model outputs were compared to annotator ratings. Another study by Andy and Guntuku [47] compared the predictive performance of random forest models developed to measure the degree of social and informational support seeking expressed in post titles. They showed that the type of social support in post titles varies according to the substance use recovery forum.

### Infoveillance Studies

In total, 7 (23%) papers were classified as an infoveillance study. Alambo et al [48] estimated the longitudinal topical correlation between substance use or mental health and COVID-19–related posts through the COVID-19 pandemic, finding that this correlation peaked after August 2020. Balsamo et al [43] traced the temporal evolution of posts from 2014 to 2018 relating to nonmedical consumption patterns, administration routes, and drug tampering. Their findings showed that mentions of heroin and hydrocodone decreased over time, mentions of buprenorphine and oxycodone remained relatively static, and mentions of fentanyl and codeine increased. El-Bassel et al [22] explored the COVID-19 period, from March to May 2020, and revealed the topics discussed in opioid-related subreddits and how they changed over time. There was a decrease in conversation on the topic referred to as “supply shutdown,” gradual reductions in discussions on MOUD experiences and access issues, and increases in conversations on the negative consequences of OUD. Sarker et al [61] used the lexical variant generation model to examine stimulant co-mention trends among people who use opioids and people using MOUD and found that the number and proportion of redditors mentioning both increased steadily over time.

Pandrekar et al [46] analyzed the main psychological categories (ie, relativity, cognitive processes, social words, drives, affect words, biological processes, percept, and informal speech) of the posts from the r/opiates subreddit between 2014 and 2017 and showed that posts on personal issues tend to receive more social support. Sarker et al [23] compared pre– and post–COVID-19 monthly discussions on drug use, treatment access, care, and withdrawal. The results showed an increase in posts discussing withdrawal, treatment, and access to care from pre– to post–COVID-19 periods. Finally, a study by Sumner et al [62] used a time series regression with the least absolute shrinkage and selection operator approach to predict opioid overdose deaths (from CDC WONDER), using Twitter and Reddit post volume as predictor variables.

### Pharmacovigilance Studies

In total, 3 (10%) studies were classified as a pharmacovigilance study. Chancellor et al [58] identified alternative drugs for treating OUD using word embeddings. The top 5 most frequently observed potential treatments were kratom, loperamide, Xanax, Valium, and Klonopin. Preiss et al [33] aimed to find medications to cope with opioid withdrawal symptoms based on observed correlations between text entities identified within posts relating to withdrawal symptoms and substances. Besides the ones already approved by the Food and Drug Administration or commonly used to treat symptoms, they found additional medicines considered potentially helpful (eg, gabapentin for body aches) and natural or home remedies (eg, ginger for nausea). Wright et al [19] evaluated the semantic proximity of individual substances to “overdose” through the years, with fentanyl being found to experience the most significant changes as it moved more closely to overdose and overdose-related terms.

Table 3 presents the selected studies, their overarching goal, the research question they address, and the NLP methods used. This can serve as a guide to help researchers studying substance use in formulating research questions that could be answered by applying NLP methods to Reddit data and to identify which NLP methods (and miscellaneous methods) to use in each case. To facilitate this process among researchers studying substance use with no expertise in NLP methods, we provide a brief description of these methods alongside examples of research questions they can answer in Multimedia Appendix 5. Full details on the data extracted for each study during the review process are provided in Multimedia Appendix 4.

**Table 3:** Overarching goals, research questions, and methods for studies applying natural language processing methods to Reddit data to investigate opioid use.

Study	Overarching goal	Research question	Methods
Adams et al [37]	Methodological	Drug term discovery	Word embedding
Akioyamen et al [38]	Methodological	Combine methods to allow users to actively navigate through topics or posts of interest	Topic modeling (LDA^a^) and sentiment analysis
Jha and Singh [40]	Methodological	Develop a tool to analyze data from Reddit and Twitter and make it available to the academic community for use	Topic modeling (LDA), sentiment analysis, and propensity score matching
Zhu and Bhat [41]	Methodological	Produce a list of euphemistic phrase candidates that are used as substitutes for target keywords corresponding to drug names	Phrase mining on a raw text corpus
Davis et al [39]	Methodological and infodemiology—prediction or classification	Predict archetypes	Archetype-based modeling and search
Wright et al [19]	Methodological and pharmacovigilance	Measure movements over time of the semantic proximity of substance-related terms to “overdose”	Diachronic word embedding
Jha and Singh [44]	Infodemiology—associations	Identify and quantify the relationship between emotional distress, physical pain, self-development, relationships, and geographic disparities versus drug addiction recovery and relapse	Semantic-based analysis and structural equation modeling
Spadaro et al [45]	Infodemiology—associations	Study potential associations between fentanyl, buprenorphine induction, and precipitated opioid withdrawal	Annotation and term-frequency matrix
Andy [42]	Infodemiology-associations and infodemiology—prediction or classification	Measure types of self-disclosures (ie, positive and negative) and social supports sought (ie, emotional and informational)	Random forest and correlation analysis
Andy and Guntuku [47]	Infodemiology-association and infodemiology—prediction or classification	Determine the relationship between the social supports expressed in the titles of posts and the number of comments they receive	Topic modeling (LDA) and sentiment analysis
Alambo et al [48]	Infodemiology—associations and infoveillance	Monitor trends of correlation between depression or substance use disorder and coronavirus posts	Word embedding, topic modeling (LDA), and correlation analysis
Balsamo et al [43]	Infodemiology—associations and infoveillance	Characterize patterns and estimate correlations between routes of administration and drug tampering	Word embedding and correlation analysis
Pandrekar et al [46]	Infodemiology—associations and infoveillance	Understand posts’ psychological categories and examine the association between posts’ attributes and the social support received	Topic modeling (LDA), semantic-based analysis, negative binomial, and Mann-Whitney U tests
Ramachandran et al [49]	Infodemiology—discovery	Unveil public opinion on the opioid epidemic	Sentiment analysis and ANOVA
Gauthier et al [50]	Infodemiology—discovery	Understand how web-based communities support recovery	Topic modeling (LDA) and thematic analysis
Chen et al [51]	Infodemiology—discovery	Examine the nature of stigma-related experience related to substance use and the salient affective and temporal factors in the use of 3 substances (including opioids)	Manual annotation and topic modeling (NMF^b^)
Graves et al [52]	Infodemiology—discovery	Identify topics discussing firsthand experiences with buprenorphine-naloxone	Manual annotation and lexical similarity filter
ElSherief et al [60]	Infodemiology—prediction or classification	Identify misinformation related to medications for opioid use disorder	Bidirectional long short-term memory
Eshleman et al [53]	Infodemiology—prediction or classification	Predict users’ propensity for seeking drug recovery interventions	Word embedding and prediction or classification: K-NN^c^, K-NN, random forests, logistic regression, and naive Bayes.
Jha et al [55]	Infodemiology—prediction or classification	Characterize addiction stages of opioid use from users’ social media posts	Word embedding and a combination of bidirectional long short-term-memory networks and conditional random fields
Lu et al [54]	Infodemiology—prediction or classification	Predict users’ transitions from casual drug discussion forums to drug recovery forums	Word embedding, binary classifier, and Cox regression
Yang et al [59]	Infodemiology—prediction or classification	Predict relapse among people who use opioids	Topic modeling (LDA), correlation analysis, sentiment analysis, and support vector machine
Yang et al [56]	Infodemiology—prediction or classification	Predict opioid use disorder and recovery among people who use opioids	Sentiment analysis, generative adversarial networks, and correlation analysis
Yao et al [57]	Infodemiology—prediction or classification	Identify posts of suicidality among people who use opioids	Word embedding, convolutional neural network (also tested logistic regression, random forest, support vector machines, FastText, recurrent neural network, and attention-based bidirectional recurrent neural network)
Chancellor et al [58]	Infodemiology—prediction or classification and pharmacovigilance	Identify messages related to opioid use recovery and alternative treatments	Binary transfer learning classifier and word embeddings
El-Bassel et al [22]	Infoveillance	Identify challenges faced by people who use opioids and how these change over time	Word embedding and topic modeling (LDA)
Sarker et al [23]	Infoveillance	Identify prescription or illegal opioid use, describe opioid treatment access and care, and withdrawal	Annotation and term-frequency matrix
Sarker et al [61]	Infoveillance	Examine stimulant comention trends among people who use opioids or receive medications for opioid use disorder.	Generate lexical variants and negatives (LexExp^d^ and NegEx^e^)
Sumner et al [62]	Infoveillance	Build a statistical model for estimating national opioid overdose deaths using multiple predictors, including data on the volume of Reddit posts mentioning heroin and synthetic opioids.	Time-series analysis (LASSO^f^)
Preiss et al [33]	Pharmacovigilance	Identify symptoms and remedies for opioid withdrawal	Word embedding and named entity recognition

^a^LDA: latent Dirichlet allocation.

^b^NMF: nonnegative matrix factorization.

^c^K-NN: K-nearest neighbors.

^c^LexExp: unsupervised lexicon expansion system.

^d^NegEx: negation detection algorithm.

^e^LASSO: least absolute shrinkage and selection operator.

### Scope of Substance Use Populations Considered and Distinction of Opioid Types

The anonymity characteristic of Reddit data prevents any definitive description of the study participants, composed of individuals contributing and responding to the posts selected for analysis. Nonetheless, participation in subreddits implies an active personal engagement with the topic, and therefore most studies in this review presuppose that the data from posts represent the language, views, and experiences of people who use opioids. For instance, the study by Andy and Guntuku [47] et al analyzed data to investigate the types of support sought by people recovering from OUD, which was assumed based on their activity in the r/OpiatesRecovery and r/suboxone subreddits. Furthermore, 4 (13%) other studies also made inferences about the drug use and recovery status of individual redditors based on the subreddits they were found to post in [53-55]. In total, 5 (17%) studies involved the manual inspection and labeling of a sample of posts to verify whether they were indicative of ongoing opioid use, recovery efforts, or a recent relapse [55-59]. Similarly, a study by Graves et al [52] involved a manual thematic categorization of 200 posts by 3 separate researchers to determine whether the posts’ contents were indicative of personal experiences of buprenorphine-naloxone use. Another study by El-Bassel et al [22] was more cautious in acknowledging that their data might not reflect the firsthand experiences of people using drugs, despite this being the purpose of their study. In all 5 studies, the posts were labeled by health or substance use researchers except for 1 study, relying on nonspecialist workers from Amazon Mechanical Turk [57].

### Limitations Mentioned by the Authors

In total, 20 (67%) studies reported limitations associated with the methods used or the results produced. The most commonly cited limitation was the uncertainty regarding the representativeness of the community of people discussing opioid use on Reddit to the broader population of people who use opioids [23,43,45,52,54,58,61,62]. A related set of limitations was the lack of information on the demographics and location of the study participants [22,46,50] and the inability to ascertain whether study participants had a clinical diagnosis of OUD [22,43,52,54,61]. Another limitation was the presumption that subreddits dedicated to opioid use and recovery would consistently provide relevant content. A manual review undertaken in the study by Yao et al [57] showed that this was not true in many instances. Another study acknowledged that relying on thematic subreddits addressing substance use disorders disregards relevant content in nondrug use subreddits [54].

### Semantic Analysis and “Opioid Synset” Development

As Reddit forums are internet-based spaces used by people looking for information, advice, or simply to share experiences, the textual variation by colloquial terms (slang), misspellings, and abbreviations is frequent. This heterogeneity imposes a challenge for NLP methods.

More recent studies have used NLP tools for automatic variant generation [23,45,61]. Some studies have used existing lists of slang terms derived from the Drug Enforcement Administration Drug Slang Code Words [63], the Drug Abuse Ontology [64], and the Urban Dictionary [37,43,48,65]. However, many of the papers have explored the potentialities of embedding words to deal with semantic variation. While some studies incorporated word embedding methods into the analysis [22,48,53-55,57], others used this methodology with the ultimate (or intermediate) goal of expanding opioid-related vocabulary [19,33,37,43,58]. In this spirit, Zhu and Bhat [41] proposed a multiword method to identify nonsingle word euphemisms for the drug use field.

Despite the accessibility of word embeddings for document identification, it is important to emphasize that the outputs require expert curation to ensure precision in the meaning of the words used. This is because word embedding outcomes do not provide synonyms but words with similar representations. For example, in the study of Chancellor et al [58], word embedding outcomes for fentanyl included morphine, heroin, or even ketamine. We systematically categorized opioid-related terms used and generated across selected studies to produce a comprehensive “opioid synset” and support research in this area (Multimedia Appendix 6).

### Ethical Considerations

Ethics approval was reported for 2 (7%) of the studies [50,52], while a further 4 (13%) studies were granted exemptions from ethics reviews [23,47,61,62]. The studies’ dissemination vehicle did not seem to determine whether they included ethical considerations. About 45% (5/11) of the conference proceedings and books and 63% (12/19) of the peer-reviewed papers did not report obtaining ethical clearance or any type of action to protect participants. From the remaining 55% (6/11) of the conference proceedings and books, 2 (33%) submitted their projects to the Institutional review board (IRB) and had them approved or exempted, 2 (33%) used strategies to protect participants, such as paraphrasing, and 2 (33%) argued the studies did not qualify for the ethics board review. Of the 37% (7/19) of the peer-reviewed papers that reported some ethical considerations to protect participants’ privacy, 4 (57%) submitted their projects to the IRB and had them approved or exempted, and 3 (43%) argued the studies did not qualify for ethics board review. The papers’ ethical considerations are available in Multimedia Appendix 4.

## Discussion

### Contributions and Potential of This Research

This paper describes the small but growing body of research applying NLP methods to analyze content from Reddit relating to opioid use. We show that the existing literature is diverse in scope, as indicated by the combination of infodemiology, infoveillance, pharmacovigilance, and methodological studies’ overarching goals. The literature is also varied in terms of NLP methods used, which include word expansion techniques, topic models, and prediction or classification methods. We found that most studies have been led by researchers with training in computer science, which was also the leading subject area associated with the platforms used for their dissemination or publication. This trend might explain the high degree of emphasis placed on the conduct of methods-driven research in the literature that demonstrates the application of NLP methods in the context of OUD, as opposed to research being primarily motivated by a theory or hypotheses specific to OUD. Indeed, while some studies showcase NLP applications to Reddit data that could directly be used toward routine public health surveillance or interventions in the context of OUD, many studies presented NLP methodological development and used OUD as an opportune case study without considering their practical use or operationalization.

Given our focus on leveraging big data and NLP methods to improve public health responses to the opioid crisis, we provide an overview of the key contributions that have been and could be achieved through further engagement with this research. Reddit has been increasingly used as a source of information to understand opioid use behaviors and their connections with other conditions. Significant problems inherent to the substance use field, including the identification and classification of participants’ membership along the OUD continuum and the longitudinal tracking of OUD-related metrics, have been robustly explored in studies, such as Jha et al [55] and Alambo et al [48].

Compared to traditional qualitative studies that rely on recruiting and interviewing participants, studies using Reddit data provide temporal flexibility, enabling the retrospective investigation of particular periods of interest, bypassing recall bias, as well as longitudinal perspectives (which are rare in the context of qualitative studies actively engaging participants). The latter can include following redditor cohorts (ie, following the same individuals over time) [53] or specific topics (including a mix of old and new redditors participating in that specific subreddit at each time point) [23]. Indeed, the studies using Reddit data use Reddit data opportunistically, while traditional qualitative studies that actively engage participants are designed to answer specific research questions. Compared to studies using traditional qualitative methods to analyze Reddit data, we found that those studies using NLP methods identified similar topics. We identified 3 studies using qualitative thematic analysis on posts of the subreddits r/Opiates and r/OpiatesRecovery, during the peak months of the COVID-19 pandemic, March to May 2020 [66-68], which could be compared with the NLP-based study included in this scoping review using data from March to May 2020, from the subreddits r/opiates, r/OpiatesRecovery, r/suboxone, and r/Methadone [22].

The qualitative study conducted by Krawczyk et al [68], which explored the impact of changes imposed by the pandemic on OUD treatment access, found that people who use opioids were concerned about OUD treatment facilities’ closure, the transition to telehealth, inconsistency between methadone daily clinic requirements and exposure to COVID-19 risks, impressions on regulation changes for MOUD, and how the pandemic was impacting treatment motivation and progress. Bunting et al [67] also qualitatively explored COVID-19 restrictions’ consequences on the daily lives of people who use opioids, such as social isolation and the consequent change in the social network, and how Reddit was used to ask for or offer advice. Finally, Arshonsky et al [66] described psychological and behavioral coping strategies for withdrawal symptoms and cessation or reduction of opioid use.

Comparatively, the paper by El-Bassel et al [22] on NLP provided an overview of the main topics representing different concerns around OUD in the context of COVID-19 and its trend and changes as the pandemic evolved. It found similar access issues during the COVID-19 months, such as closed and overcrowded treatment centers, limited take-home doses, switching from methadone to buprenorphine treatment to avoid daily clinic visits, or switching from oral to injectable buprenorphine to avoid access barriers, financial barriers to access MOUD, helpfulness of telehealth, withdrawal issues originated by diminished drug supply and MOUD facilities’ closures, confusion if their symptoms were because of withdrawal or COVID-19 infection, stigmatization by health care providers, self-medication to keep in the recovery track, and home detox. Sarker et al [23] also found similar topics during the COVID-19 pandemic to those reported by El-Bassel et al [22] and by the 3 qualitative studies mentioned [66-68], with the bonus of following the relative frequency of themes associated with drug use, treatment and access, and withdrawal over time (ie, infoveillance). This suggests NLP methods can effectively identify key themes while saving time and covering larger textual corpora. A study purposefully designed to compare NLP versus traditional qualitative methods is needed to rigorously investigate the advantages and drawbacks of NLP methods.

Given the complexity of the research questions, in general, researchers used a mix of NLP methods to address opioid-related problems. Traditional NLP methods, such as topic modeling, were used more frequently to discover aspects of opioid use behavior and follow them over time (infodemiology and infoveillance papers), whereas word embedding was often used to discover new drugs (pharmacovigilance) and in tandem with classification and prediction methods to detect people’s stage in the OUD continuum (infodemiology). In contrast, sentiment analysis was mostly used to infer redditor’s opinions and positioning about opioid use. Another important aspect of the studies analyzed in this scoping review is its implications for public health responses. While none of the studies described the implementation of Reddit-based interventions, several studies have the potential to be used toward such purposes. For example, the study by ElSherief et al [60] aimed to identify misinformation related to MOUD. It is natural to foresee how such a tool could be used on a routine basis to detect existing and new myths, and both respond to those posts providing evidence-based information as well as to compile these myths to guide public health messaging by providers and relevant agencies. Studies presenting methods to identify users at high risk of relapse [55] or suicide [57] could also be used to reach out to these individuals with helpful resources, including crisis lines and other types of support, as has been done in other studies [69-71]. Similarly, those identified as having a high probability of transitioning to an opioid recovery subreddit could benefit from information and resources to engage in treatment, counseling, support groups, or activities to facilitate this process [53].

### Methodological Remarks and Recommendations

The NLP methods used by the selected studies were diverse. We provided a map to identify appropriate methods depending on the research problem addressed. In addition, we have endeavored to bring clarity regarding the different types of data used in the studies. The commonly used term *post*, used across many studies is not specific enough as it does not fully characterize the data considered (first submission, comment, or whether it includes the title). Similarly, it is not always clear how many posts were included in the analyses (eg, all those available over a period versus a sample of the posts submitted) and how many different users these posts represented. Importantly, details about the subreddits considered and the period covered are not systematically specified. Best practices for Reddit or other social media platforms’ data description should include these key characteristics to improve transparency about the representativity of the data and to enable its reproducibility.

We also found important variation in terms of the use of “dictionaries” to enable the identification of opioid use–related content, which can have an impact on the results through modifying the body of data examined. We, therefore, offer an important resource to increase the quality of new research on opioid use undertaken in this space through the synset we compiled and shared in Multimedia Appendix 6.

Regarding the use of NLP on Reddit data, some considerations should be highlighted. Approaching Reddit data from an exploratory perspective, at least initially, is useful to ascertain the extent to which Reddit data are suitable for your particular research question. Careful selection of appropriate subreddits is important given that each subreddit has a distinct culture and associated norms. For example, the r/trees subreddit is devoted to the discussion of cannabis use, whereas the r/marijuanaenthusiasts subreddit is devoted to the discussion of arboreal matters. Reddit-based research—particularly in health-related areas—should be assessed by research ethics committees or IRBs and follow research ethics protocols related to privacy protection. For example, Benton et al [72] recommend adopting policies related to privacy protection (eg, refraining from using direct quotations in presentations and publications) and—where appropriate—deidentifying data (eg, using anonymous numerical codes in the place of social media usernames).

Recent changes in Reddit’s application programming interface access have the potential to affect future research that relies on real-time, high-throughput data access. However, historical data (before April 2023) remains publicly available via PushShift and the free Reddit application programming interface (accessed via PRAW in Python) can be used to collect recent posts containing specific keywords. Furthermore, it is possible to purchase data from Reddit, with the study by Poudel and Weninger [73] estimating a potential cost of US $240 per 1 million posts. Finally, as of late May 2024, Reddit is planning on implementing a new service aimed at providing academic researchers with affordable data [74].

Given the rapid changes in NLP technology, it is difficult to predict the future directions of application of NLP methods to address substance use disorders–related research questions, particularly concerning the future performance of large language models such as Gemini and ChatGPT (OpenAI LP). However, 2 broad themes are emerging. First, a growing democratization of NLP methods, given that generative NLP methods allow for the creation of baseline NLP tools by public health experts with relatively little computer science expertise (for example, by using prompt engineering approaches). Second, potentially increasing costs associated with accessing data for research given the industry-wide drive to monetize social media data.

### Limitations

Our findings highlight 2 fundamental paradoxes associated with NLP-based research on opioids using content from Reddit. The first paradox relates to the anonymity afforded to Reddit users to disclose their own experiences and viewpoints related to opioid use. While this anonymity is advantageous as far as it supports the availability of evidence offering unique insights compared to other sources (eg, routine household surveys), it also represents a limitation because it implies a lack of demographic and clinical information on study participants. This may cast doubts upon the authenticity of the findings as there is no way of verifying whether the content reflects firsthand experiences or whether study participants meet the clinical criteria for a diagnosis of OUD. The second paradox relates to the methodological capabilities of NLP techniques to draw inferences from user-generated textual data. These computational methods have facilitated the large-scale computation of specific tasks, such as content identification and detection of textual patterns involving huge quantities of data. Despite these advances, there appear to be limits on the types of inferences that NLP methods can make, particularly when it comes to analyses that go beyond content identification (eg, posts related to opioid use) and attempt to interpret meaning. This observation corresponds with findings elsewhere, showing that NLP methods are valuable adjuncts to traditional qualitative research methods but do not yet represent an adequate alternative to human analyses [75].

Despite the extensive use of bespoke lexica to perform analysis of social media text, these lexicon-based approaches remain error-prone due to ambiguities characteristic of natural language. For example, different drugs may be referred to by the same slang term in different contexts (eg, “junk” can be used to refer to either heroin or cocaine).

A methodological limitation of this scoping review is the inclusion of papers exclusively written in English. Although more than half of Reddit users are from countries with the ongoing opioid epidemic and that speak predominantly English, such as the United States, United Kingdom, Canada, and Australia [24], this imposes a potential constrain on the understanding of how researchers are using NLP methods in Reddit to investigate opioid use.

### Conclusions

This comprehensive review explores the expanding application of NLP methodologies to analyze text data sourced from Reddit, with a particular focus on opioid-related subreddits. A wide variety of NLP techniques and applications can be observed in the literature that demonstrate the potential for NLP use to support surveillance and social media interventions addressing the opioid crisis. Although we found that these methods offer useful insights into the behavior and attitudes of people who use opioids, there are limits to the utility of these automated approaches, with current methods best thought of as supplementary to current, established epidemiological methods.

## Appendix

Multimedia Appendix 1 Joanna Briggs Institute population (or participants)/concept/context (PCC) framework rationale behind each research question.

Multimedia Appendix 2 Search terms strategy.

Multimedia Appendix 3 PRISMA-ScR (Preferred Reporting Items for Systematic Reviews and Meta-Analyses Extension for Scoping Reviews) checklist.

Multimedia Appendix 4 Data extraction table.

Multimedia Appendix 5 Brief definitions of the natural language processing methods most used by the scoping review papers, according to Wikipedia (accessed on January 20, 2023).

Multimedia Appendix 6 Synset.

## Abbreviations

DAR: drug addiction recovery
IRB: institutional review board
JBI: Joanna Briggs Institute
MOUD: medication for opioid use disorder
NLP: natural language processing
OUD: opioid use disorder
PRISMA: Preferred Reporting Items for Systematic Reviews and Meta-Analyses
PRISMA-ScR: Preferred Reporting Items for Systematic Reviews and Meta-Analyses Extension for Scoping Reviews
