# Supplementary material for: The Use of Natural Language Processing Methods in Reddit to Investigate Opioid Use: Scoping Review
Source: JMIR Infodemiology. 2024 Sep 13;4:e51156. doi: 10.2196/51156 (PMC11437337; doi:10.2196/51156)
Supplement: Multimedia Appendix 1 [file infodemiology_v4i1e51156_app1.docx]

Multimedia Appendix 1: Joanna Briggs Institute PCC framework rationale behind each research question

- Question 1: What are the main opioid use-related research questions approached by applying natural language processing methods to Reddit data?

o Population: People who use opioids

o Concept: opioid use-related research questions approached through natural language processing methods

o Context: Reddit forums

- Question 2: What are the main opioid use-related studies’ overarching goals/key objectives pursued by natural language processing methods using Reddit data?

o Population: People who use opioids

o Concept: Opioid use-related studies’ overarching goals/key objectives approached by natural language processing methods

o Context: Reddit forums

- Question 3: What are the main natural language processing methodologies applied in opioid use-related studies using Reddit data?

o Population: People who use opioids

o Concept: natural language processing methodologies applied in opioid use-related studies

o Context: Reddit forums

- Question 4: Which softwares are used to apply NLP methods to answer opioid use-related research questions using Reddit data?

o Population: People who use opioids

o Concept: Softwares used to apply natural language processing methods to answer opioid use-related research questions

o Context: Reddit forums

- Question 5: What are the main opioid use-related studies’ limitations when using natural language processing methods in Reddit data?

o Population: People who use opioids

o Concept: Opioid use-related studies’ limitations when using natural language processing methods

o Context: Reddit forums
