# Supplementary material for: The Use of Natural Language Processing Methods in Reddit to Investigate Opioid Use: Scoping Review
Source: JMIR Infodemiology. 2024 Sep 13;4:e51156. doi: 10.2196/51156 (PMC11437337; doi:10.2196/51156)
Supplement: Multimedia Appendix 2 [file infodemiology_v4i1e51156_app2.docx]

Multimedia Appendix 2: Search terms strategy

Below, we present the Medical Subject Headings (MeSH) vocabulary thesaurus used for indexing AI articles provided by the National Library of Medicine, followed by the final query used.


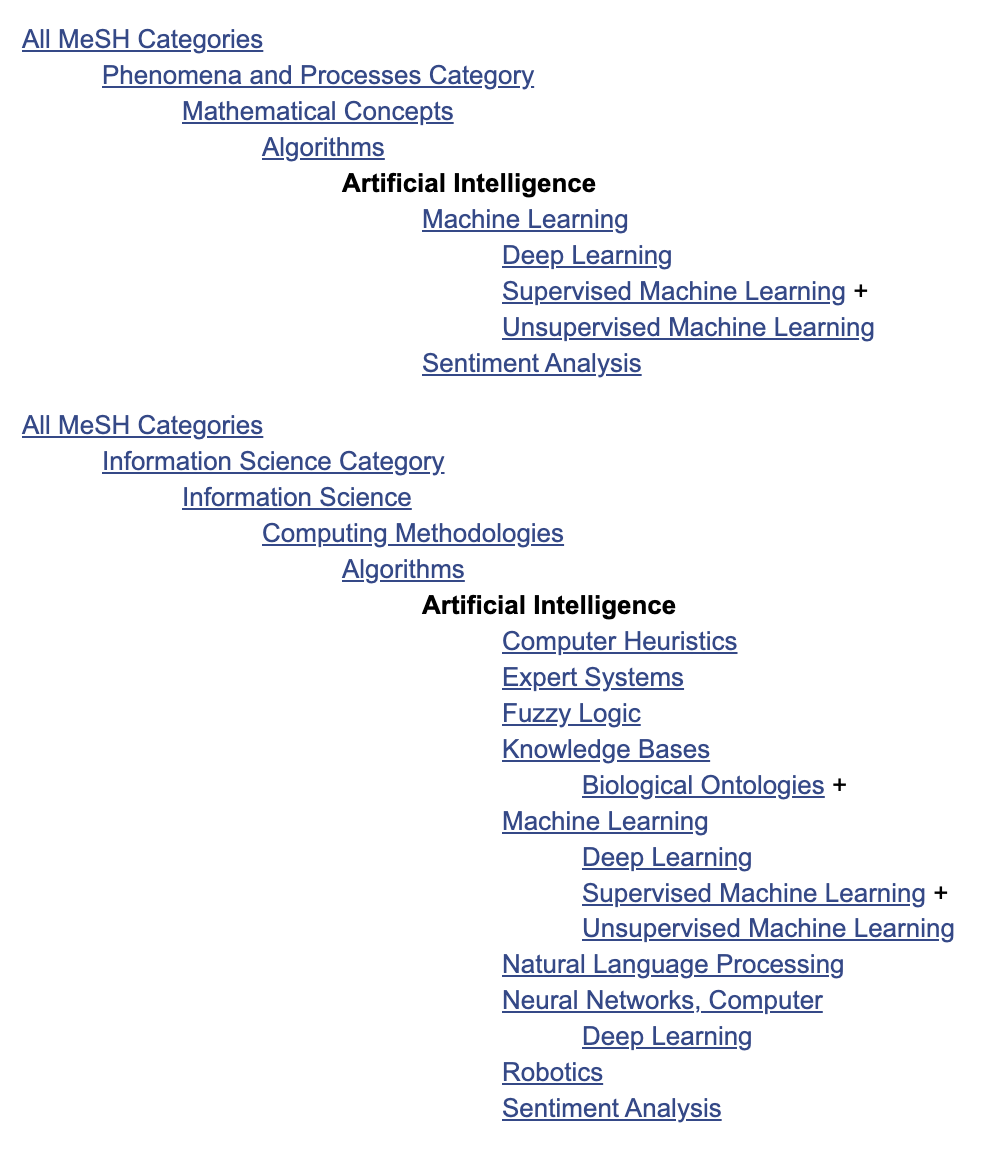


Source: <https://www.ncbi.nlm.nih.gov/mesh/68001185>

Final query used (the first block relates to opioid use, the second to the methodology, and the third the social media aspect):

**(** (Opioid* OR Opiate* OR Heroin OR Buprenorphine OR Suboxone OR Subutex OR Methadone OR Naltrexone OR Vivitrol OR Hydrocodone OR Vicodin OR Oxycodone OR OxyContin OR Percocet OR Oxymorphone OR Opana OR Morphine OR Kadian OR Avinza OR Codeine OR Fentanyl OR MMT OR “Medication Maintenance Treatment” OR “Medication Maintenance Therapy” OR MAT OR “Medication-Assisted Treatment” OR “Medication-Assisted Therapy”)

AND

(“Artificial Intelligence” OR “Machine Learning” OR ML OR “Deep learning” OR “Computer Heuristics” OR “Expert Systems” OR “Knowledge Bases” OR ontolog* OR robotics OR “Sentiment Analysis” OR “Support Vector Machine” OR “Fuzzy Logic” OR “Natural Language” OR NLP OR “Neural network” OR “Generative Adversarial Network” OR “Text Mining” OR Semiautomatic OR “Topic Model*” OR “Transfer Learning”)

AND

(“reddit” OR “social media”) **)**
