# Supplementary material for: The Use of Natural Language Processing Methods in Reddit to Investigate Opioid Use: Scoping Review
Source: JMIR Infodemiology. 2024 Sep 13;4:e51156. doi: 10.2196/51156 (PMC11437337; doi:10.2196/51156)
Supplement: Multimedia Appendix 5 [file infodemiology_v4i1e51156_app5.docx]

Multimedia Appendix 4: Brief definitions of the NLP methods most used by the scoping review papers, according to Wikipedia (accessed on January 20th, 2023)

| **NLP method** | **Definition** | **Examples of research question addressed in this scoping review** | **Examples of papers in this scoping review using the method** | **Specificities of the methods used in the examples** |
| --- | --- | --- | --- | --- |
| Word embedding | A word embedding is a representation of a word. Typically, the representation is a real-valued vector that encodes the meaning of the word in such a way that words that are closer in the vector space are expected to be similar in meaning. Word embeddings can be obtained using language modeling and feature learning techniques where words or phrases from the vocabulary are mapped to vectors of real numbers. (https://en.wikipedia.org/wiki/Word_embedding) | Drug term discovery, identification of documents with similar content, and analysis of words’ semantic change over time. | Choosing Your Platform for Social Media Drug Research and Improving Your Keyword Filter List (Adams et al., 2019) | Word2Vec |
|  |  |  | Detection of emerging drugs involved in overdose via diachronic word embeddings of substances discussed on social media (Wright et al., 2021) | Word2Vec |
| Topic Modelling | Type of statistical model for discovering the abstract "topics" that occur in a collection of documents. Topic modeling is a frequently used text-mining tool for discovery of hidden semantic structures in a text body. Intuitively, given that a document is about a particular topic, one would expect particular words to appear in the document more or less frequently: "dog" and "bone" will appear more often in documents about dogs, "cat" and "meow" will appear in documents about cats, and "the" and "is" will appear approximately equally in both. A document typically concerns multiple topics in different proportions; thus, in a document that is 10% about cats and 90% about dogs, there would probably be about 9 times more dog words than cat words. (https://en.wikipedia.org/wiki/Topic_model) | Discovery of themes within a set of documents. | “I Will Not Drink With You Today”: A Topic-Guided Thematic Analysis of Addiction Recovery on Reddit (Gauthier et al., 2022) | LDA with 16-topic solution |
|  |  |  | Harnessing the Power of Social Media to Understand the Impact of COVID-19 on People Who Use Drugs During Lockdown and Social Distancing (El-Bassel et al., 2022) | Latent feature topic models (LF-LDA) with lambda=0.6 and 10-topic solution. |
| Sentiment analysis | Also known as opinion mining or emotion NLP, it is the use of natural language processing, text analysis, computational linguistics, and biometrics to systematically identify, extract, quantify, and study affective states and subjective information. A basic task in sentiment analysis is classifying the polarity of a given text at the document, sentence, or feature/aspect level—whether the expressed opinion in a document, a sentence or an entity feature/aspect is positive, negative, or neutral. Advanced, "beyond polarity" sentiment classification looks, for instance, at emotional states such as enjoyment, anger, disgust, sadness, fear, and surprise (https://en.wikipedia.org/wiki/Sentiment_analysis). | Identification of emotional/psychological tones within a set of documents. | Tones and themes in Reddits posts discussing the opioid epidemic (Ramachandran et al., 2022) | IBM Watson NLU, a  deep learning tool to extract meaning and metadata  from unstructured text data. |
|  |  |  | A Framework for Intelligent Navigation Using Latent Dirichlet Allocation on Reddit Posts About Opiates (Akioyamen et al., 2020) | NRC emotion lexicon and the AFINN  Lexicon. |
| Prediction/ classification | Usually uses more than one method and comparing the results. The most popular among the papers analyzed in this scoping review was the Support Vector Machine (SVM). SVM are supervised learning models with associated learning algorithms that analyze data for classification and regression analysis. an SVM training algorithm builds a model that assigns new examples to one category or the other, making it a non-probabilistic binary linear classifier (although methods such as Platt scaling exist to use SVM in a probabilistic classification setting). SVM maps training examples to points in space so as to maximize the width of the gap between the two categories. New examples are then mapped into that same space and predicted to belong to a category based on which side of the gap they fall. (https://en.wikipedia.org/wiki/Support_vector_machine). | Prediction of Redditors characteristics or subreddits engagement based on previous data training. | Opioid Relapse Prediction with GAN (Yang et al., 2019) | Support vector machine |
|  |  |  | Identifying Individuals Amenable to Drug Recovery Interventions through Computational Analysis of Addiction Content in Social Media (Eshleman et al., 2017) | K-NN (3), K-NN (11), random forests, logistic regression, and naive Bayes. |
